# PP62 Assessment Of Evidence On The Efficacy And Safety Of Oral Nutritional Supplements For Malnutrition In Hospitals

**DOI:** 10.1017/S026646232510281X

**Published:** 2025-12-29

**Authors:** Keyla Caroline Almeida, Everton Macêdo Silva, Larissa Silva, Juliana Girardi

## Abstract

**Introduction:**

Hospital malnutrition is caused by metabolic imbalance and is associated with increased rates of morbidity and mortality and unfavorable prognosis for hospitalized patients. In 2001, severe malnutrition was detected in 12.5 percent of hospitalized patients in Brazil. The aim of this study was to evaluate evidence on the efficacy and safety of oral nutritional supplements for malnutrition in hospitalized patients.

**Methods:**

Search strategies developed for the PubMed, Embase, LILACS and Cochrane Library databases were used to search and select the available evidence. The included evidence compared oral nutritional supplementation with standard hospital care or placebo. A total of 47 clinical trials were selected. For mortality, a meta-analysis was performed combining the effect measures of 40 clinical trials. R software was used with the meta package installed. The revised Cochrane risk-of-bias tool was used to assess the risk of bias.

**Results:**

For the primary outcomes of mortality (relative risk [RR] 0.94, 95% CI: 0.77, 1.15), length of hospital stay, and number of hospital readmissions (RR 0.97, 95% CI: 0.74, 1.28), oral supplementation offered no benefit, compared with the control group. Regarding infectious and non-infectious complications, there was a significant reduction in the number of patients with complications in the supplemented group, compared with the control group (RR 0.71, 95% CI: 0.59, 0.86). Regarding the risk-of-bias assessment, most of the studies had a high risk of bias and limitations in almost all areas.

**Conclusions:**

The designs of the selected studies were quite heterogeneous in terms of follow-up time, population, and sampling, among others, and several doubts were raised about the methodology used in the clinical trials. These aspects were reflected in the low quality and high risk of bias of the primary studies, which proved to be an important limitation.

